# Seasonal Effect on the Chemical Composition, Insecticidal Properties and Other Biological Activities of *Zanthoxylum leprieurii* Guill. & Perr. Essential Oils

**DOI:** 10.3390/foods9050550

**Published:** 2020-05-01

**Authors:** Evelyne Amenan Tanoh, Guy Blanchard Boué, Fatimata Nea, Manon Genva, Esse Leon Wognin, Allison Ledoux, Henri Martin, Zanahi Felix Tonzibo, Michel Frederich, Marie-Laure Fauconnier

**Affiliations:** 1 Laboratory of Biological Organic Chemistry, UFR-SSMT, University Felix Houphouet-Boigny, 01 BP 582 Abidjan 01, Ivory Coast; blancardo2000@yahoo.fr (G.B.B.); neafatima@gmail.com (F.N.); tonzibz@yahoo.fr (Z.F.T.); 2 Laboratory of Chemistry of Natural Molecules, University of Liège, Gembloux Agro-Bio Tech, 2, Passage des Déportés, 5030 Gembloux, Belgium; m.genva@uliege.be (M.G.); henri.martin@uliege.be (H.M.); marie-laure.fauconnier@uliege.be (M.-L.F.); 3 Laboratory of Instrumentation Image and Spectroscopy, National Polytechnic Institute Felix Houphouët-Boigny, BP 1093 Yamoussoukro, Ivory Coast; esse.wognin@yahoo.fr; 4 Laboratory of Pharmacognosy, Center for Interdisciplinary Research on Medicines (CIRM), University of Liège, Avenue Hippocrate 15, 4000 Liège, Belgium; allison.ledoux@uliege.be (A.L.); m.frederich@uliege.be (M.F.)

**Keywords:** *Zanthoxylum leprieurii*, essential oils, *Sitophilus granarius*, tridecan-2-one, β-myrcene, (*E*)-β-ocimene, dendrolasin, antioxidant, anti-inflammatory, insecticidal, anti-plasmodial, Côte d’Ivoire

## Abstract

This study focused, for the first time, on the evaluation of the seasonal effect on the chemical composition and biological activities of essential oils hydrodistillated from leaves, trunk bark and fruits of *Zanthoxylum leprieurii* (*Z. leprieurii*), a traditional medicinal wild plant growing in Côte d’Ivoire. The essential oils were obtained by hydrodistillation from fresh organs of *Z. leprieurii* growing on the same site over several months using a Clevenger-type apparatus and analyzed by gas chromatography-mass spectrometry (GC/MS). Leaf essential oils were dominated by tridecan-2-one (9.00 ± 0.02–36.80 ± 0.06%), (*E*)-β-ocimene (1.30 ± 0.50–23.57 ± 0.47%), β-caryophyllene (7.00 ± 1.02–19.85 ± 0.48%), dendrolasin (1.79 ± 0.08–16.40 ± 0.85%) and undecan-2-one (1.20 ± 0.03–8.51 ± 0.35%). Fruit essential oils were rich in β-myrcene (16.40 ± 0.91–48.27 ± 0.26%), citronellol (1.90 ± 0.02–28.24 ± 0.10%) and geranial (5.30 ± 0.53–12.50 ± 0.47%). Tridecan-2-one (45.26 ± 0.96–78.80 ± 0.55%), β-caryophyllene (1.80 ± 0.23–13.20 ± 0.33%), α-humulene (4.30 ± 1.09–12.73 ± 1.41%) and tridecan-2-ol (2.23 ± 0.17–10.10 ± 0.61%) were identified as major components of trunk bark oils. Statistical analyses of essential oil compositions showed that the variability mainly comes from the organs. Indeed, principal component analysis (PCA) and hierarchical cluster analysis (HCA) allowed us to cluster the samples into three groups, each one consisting of one different *Z. leprieurii* organ, showing that essential oils hydrodistillated from the different organs do not display the same chemical composition. However, significant differences in essential oil compositions for the same organ were highlighted during the studied period, showing the impact of the seasonal effect on essential oil compositions. Biological activities of the produced essential oils were also investigated. Essential oils exhibited high insecticidal activities against *Sitophilus granarius*, as well as antioxidant, anti-inflammatory and moderate anti-plasmodial properties.

## 1. Introduction

*Zanthoxylum leprieurii* Guill and Perr. (syn. *Fagara leprieurii* Engl and *Fagara Angolensis*) is a plant species belonging to the Genus *Zanthoxylum* of the Rutaceae family, which contains approximatively 150 Genus and 900 species. *Z. leprieurii* is distributed in rain forests and wooded savannahs in Africa, from Senegal (Western Africa), Ethiopia (Eastern Africa), to Angola, Zimbabwe and Mozambique (Southern Africa) [1]. Known as a multipurpose species, *Z. leprieurii* has a wide spectrum of applications, as leaves, trunk bark and roots are used in traditional medicine to cure rheumatism and for the treatment of tuberculosis and generalized body pains in Central and Western Africa [2,3,4]. Roots are used as chewing sticks to clean the mouth [5]. Moreover, this plant is also used for canoes, boxes, plywood, general carpentry, domestic utensils, beehives and water pots; the pale yellow wood is tough, medium coarse-grained and light [6]. Dried fruits of *Z. leprieurii* are used as spices by local populations in many regions of Africa [7]. The plant has shown antioxidant, antimicrobial, anticancer, cytotoxic, schistosomidal and antibacterial properties [8,9,10,11,12]. From a chemical point of view, a large variety of compounds from different chemical classes were reported in *Z. leprieurii* solvent extracts: acridone alkaloids, benzophenanthridine [13], aliphatic amide [14], coumarins [15] and kaurane diterpenes [11]. Essential oils produced from *Z. leprieurii* revealed that the main constituents in fruit essential oils were (*E*)-β-ocimene (29.40%) and β-citronellol (17.37%) [15,16,17]. Our previous study showed the predominance of tridecan-2-one (47.50%) in leaf essential oils from Côte d’Ivoire [18]. Limonene (94.90%) and terpinolene (50.00%) were described as major components in essential oils from Nigeria and Cameroon, respectively [19,20]. The composition of trunk bark essential oils was predominated by sesquiterpenoids in Nigeria and methyl ketones in Côte d’Ivoire [18,19].

All these studies highlighted significant differences in the composition of essential oils extracted from different organs of *Z. leprieurii* from the same growing site, as well as significant differences in the composition of essential oils extracted from *Z. leprieurii* growing in different places. The latter can be explained by the fact that many factors affect essential oil amounts as well as their chemical compositions [21,22,23]. These include plant genetic differences, as well as environmental factors such as temperature, soil, precipitation, wind speed, rainfall and pests [24].

The first aim of this study was to explore climate-related variations of the composition of essential oils hydrodistillated from different *Z. leprieurii* organs (leaves, trunk bark and fruits). The variation over time of essential oil chemical compositions was then studied at one site in Côte d’Ivoire. GC/MS was used to identify essential oil chemical profiles and statistical analyses were performed on the different chemical compositions. In addition, this study also aimed to evaluate the impact of *Z. leprieurii* essential oil composition variations on their in vitro biological activities, such as antioxidant, anti-inflammatory, insecticidal and anti-plasmodial activities. To our knowledge, such studies have not yet been carried out on this species in Côte d′Ivoire.

## 2. Materials and Methods

### 2.1. Plant Material and Hydrodistillation Procedure

*Z. leprieurii* organs were collected at Adzope (6°06′25” N, 3°51′36” W), in south-eastern Côte d’Ivoire, between May and November 2017 for leaves and trunk bark, respectively, and between July and November 2017 for fruits. At each harvest, leaf, fruit and trunk bark samples were taken from 15 randomly selected trees, in the geographical area described before, by taking the same amount of plant material from each tree and pooling it before distillation. The total amount of plant material was between 700 and 1500 g. The same tree was only sampled once to prevent the previous sampling from influencing the next one (e.g., trunk injury). Plants were identified by the Centre Suisse of Research (Adiopodoumé, Abidjan, Côte d’Ivoire) and by the National Flora Center (CNF; Abidjan, Côte d’Ivoire). The vouchers of the specimen (UCJ016132) have been deposited at the CNF Herbarium. Fresh organ material was hydrodistillated for 3 h using a Clevenger-type apparatus. The pale yellow essential oils were treated with anhydrous sodium sulphate (Na_2_SO_4_) as a drying agent, stored in sealed amber vials, and conserved at 4 °C before analysis. The essential oil yields (*w/w*) were calculated as the rapport between the mass of essential oils obtained compared to the mass of fresh organs. 

### 2.2. GC/MS Chemical Analysis of Essential Oils

Essential oils hydrodistillated from *Z. leprieurii* organs were analyzed by GC/MS. An Agilent GC system 7890B (Agilent, Santa Clara, CA, USA) equipped with a split/splitless injector and an Agilent MSD 5977B detector was used. The experience was repeated three times for each essential oil. One µL of essential oil dilutions (0.01% in hexane; *w/v*) was injected in splitless mode at 300 °C on an HP-5MS capillary column (30 m × 0.25 mm, d*f* = 0.25µm). The temperature was maintained one min at 50 °C, and then increased at a rate of 5 °C/min until 300 °C. The final temperature was maintained for 5 min. The sources and quadrupole temperatures were fixed at 230 °C and 150 °C, respectively. The scan range was 40–400 m/z, and the carrier gas was helium at a flow rate of 1.2 mL/min. The component identification was performed on the basis of chromatographic retention indices (RI) and by comparison of the recorded spectra with a computed data library (Pal 600K^®^) [25,26,27]. RI values were measured on an HP-5MS column (Agilent, Santa Clara, CA, USA). RI calculations were performed in temperature program mode according to a mixture of homologues n-alkanes (C7–C30), which were analyzed under the same chromatographic conditions. The main components were confirmed by comparison of their retention and MS spectrum data with co-injected pure references (Sigma, Darmstadt, Germany) when commercially available.

### 2.3. Biological Activities

#### 2.3.1. Antioxidant Assay

##### 2,2-Diphenyl-1-Picrylhydrazyl (DPPH) Radical Scavenging Capacity

The hydrogen atom- or electron-donating ability of essential oils and Trolox was determined from the bleaching of the purple-colored methanol DPPH solution. Briefly, the samples were tested at 25, 50, 75 and 100 µg/mL. Ten microliters of various concentrations (1 to 5 mg/mL) of each essential oil in methanol were added to 1990 μL of a 10 mg/mL DPPH methanol solution (0.06 mM). Free radical scavenging activities of leaf, trunk bark and fruit essential oils hydrodistillated from *Z. leprieurii* were determined spectrophotometrically [28]. The mixture was vortexed for about 1 min and then incubated at room temperature in the dark for 30 min; absorbance was measured at 517 nm with an Ultrospec UV-visible, dual beam spectrophotometer (GE Healthcare, Cambridge, UK). The same sample procedure was followed for Trolox (Sigma, Darmstadt, Germany) used as standard; methanol (Sigma, Darmstadt, Germany) with DPPH was used as control; and all the samples were tested in triplicate. The optical density was recorded, and the inhibition percentage was calculated using the formula given below:

Inhibition percentage of DPPH activity (%) = (Abs Blank-Abs sample)/(Abs blank) × 100(1)

Abs Blank = absorbance of the blank sample, Abs sample = absorbance of the test sample

##### Ferric-Reducing Power Assay

The ferric-reducing antioxidant power (FRAP) of essential oils hydrodistillated from *Z. leprieurii* was determined here. Briefly, four dilutions of essential oils and Trolox were prepared in methanol (25, 50, 75 and 100 µg/mL). Trolox was used as the standard reference. One mL of those methanol solutions were melded with one mL of a phosphate buffer (0.2 M, pH = 6.6) and with one mL of a potassium ferricyanide solution (1%; K_3_Fe(CN)_6_). After 20 min at 50 °C and the addition of one mL of trichloroacetic acid (TCA; 10% *v/v*), the solution was centrifuged at 3000 rpm for 10 min [27,28,29]. Next, 1.5 mL of the upper phase was recovered and melded with 1.5 mL of distillated water and 150 µL of FeCl_3_ (0.1% *v/v*). Each concentration was realized as triplicated. Finally, the absorbance of the prepared sample was measured at 700 nm. In comparison with the blank, a higher absorbance shows a high reducing power. For all concentrations, absorbance due to essential oil samples were removed from each measurement.

#### 2.3.2. Anti-Inflammatory Activity

##### Inhibition Lipoxygenase Assay

The anti-inflammatory activities of *Z. leprieurii* essential oils were determined by the method previously described by Nikhila [30]. In brief, the reaction mixture containing essential oils in various concentrations (100, 75, 50 and 25 µg/mL of methanol) (in triplicate for each concentration), lipoxygenase (Sigma, Darmstadt, Germany) and 35 µL (0.1 mg/mL) of a 0.2 M borate buffer solution (pH = 9.0) was incubated for 15 min at 25 °C. The reaction was then initiated by the addition of 35 µL of a substrate solution (linoleic acid 250 µM), and the absorbance was measured at 234 nm. Quercetin (Sigma, Darmstadt, Germany) was used as a standard inhibitor at the same concentration as the essential oils. The inhibition percentage of lipoxygenase activity was calculated as follows:

Inhibition percentage % = (Abs Blank-Abs sample)/(Abs blank) × 100(2)

where Abs blank is the Absorbance (Abs) of the reaction media without the essential oil, and Absorbance sample is the Abs of the reaction media with the essential oil minus the Abs value of the diluted essential oil (to compensate for absorbance due to the essential oils themselves).

##### Inhibition of Albumin Denaturation Assay

This test was conducted as described by Kar [31] with some slight modifications. The reaction mixture consisted of 1 mL essential oil samples and diclofenac (standard) at 100, 75, 50 and 25 µg/mL in methanol, 0.5 mL bovine serum albumin (BSA) at 2% in water and 2.5 mL phosphate-buffered saline adjusted with hydrochloric acid (HCl) to pH 6.3. The tubes were incubated for 20 min at room temperature, then heated to 70 °C for 5 min and subsequently cooled for 10 min [32]. The absorbance of these solutions was determined using a spectrophotometer at 660 nm. The experiment was performed in triplicate. The inhibition percentage of albumin denaturation was calculated on a percentage basis relative to the control using the formula:

Inhibition percentage of denaturation % = (Abs Blank-Abs sample)/(Abs blank) × 100(3)

where Abs blank corresponds to the Absorbance (Abs) of the reaction media without the addition of essential oil. Absorbance sample is the Abs of the reaction media with addition of essential oil, subtracted by the Abs value of the diluted essential oil (to compensate for absorbance due to the essential oils themselves).

IC_50_ (half inhibitory concentration), which corresponds to the essential oil concentration needed to inhibit 50% of the activity, was used to express antioxidant and anti-inflammatory properties of essential oils.

#### 2.3.3. Insecticidal Activity

##### Determination of Mortality Values

Essential oil dilutions (10, 14, 18, 22, 26 and 30 µL/mL) were prepared in acetone. Talisma UL (Biosix, Hermalle-sous-Huy, Belgium), a classical chemical insecticide used for the protection of stored grains against insects, was also used at the same concentrations. For each test, 500 µL of essential oil or standard solution were homogeneously dispersed in tubes containing 20 g of organic wheat grains. The solvent was allowed to evaporate from grains for 20 min before infesting them by 12 adult insects. The granary weevil, *Sitophilus granarius*, was chosen for this study because it is one of the most damaging cereal pests in the world. Moreover, this insect is a primary pest, which means it is able to drill holes in grains, laying its eggs inside them and allowing secondary pests to develop in the grains [33,34]. Acetone was used as a negative control. Six replicates were created for all treatments and controls, and they were incubated at 30 °C. The mortality was recorded after 24 h of incubation. Results from all replicates were subjected to Probit Analysis using Python 3.7 program to determine LC_50_, LC_90_ and LC_95_ values.

##### Repulsive Assay

This test has been conducted to evaluate the repulsive effect of essential oils against insects (*Sitophilus granarius*). This experiment was carried out by cutting an 8 cm diameter filter paper in half. The six concentrations (10, 14, 18, 22, 26 and 30 µL/mL) of essential oils were prepared in acetone. Each half disk was treated with 100 µL of the solution, and the other half with acetone. After evaporation of acetone, the two treated parts were joined together by an adhesive tape and placed in a petri dish. Ten insects were placed in the center of each petri dish and were incubated at 30 °C. After two hours of incubation, the number of insects present in the part treated with essential oil and the number of insects present in the part treated only with acetone were counted, as described by Mc Donald [35].

The percentage of repulsively was calculated as follows:

Pr = (Nc-NT)/(Nc+NT) × 100(4)

NC: Number of insects present on the disc part treated with acetone; NT: Number of insects present on the part of the disc treated with the essential oil dilution in acetone

#### 2.3.4. Anti-Plasmodial Activity

Ledoux et al. method [36] was used to determine anti-plasmodial activity. To do so, asexual erythrocyte stages of *P. falciparum*, chloroquine-sensitive strain 3D7 were continuously cultivated in vitro using the procedure of Trager and Jensen. The erythrocyte had been initially obtained from a patient from Schipol in the Netherlands (BEI Reagent Search) [37]. ATCC, Bei Ressources provides us with the strains. Red blood cells of A+ group were used as human host cell. The culture medium was RPMI 1640 from Gibco, Fisher Scientific (Loughborough, UK) composed of NaHCO_3_ (32 mM), HEPES (25 mM) and L-glutamine. Glucose (1.76 g/L) from Sigma-Aldrich (Machelen, Belgium), hypoxanthine (44 mg/mL) from Sigma-Aldrich (Machelen, Belgium), gentamin (100 mg/mL) from Gibco Fisher Scientific (Loughborough, UK) and human pooled serum from A+ group (10%) were added to the medium according to [36,38]. DMSO solutions of essential oils were directly diluted in the medium. The dilutions were performed in triplicate by successive two-fold dilutions in a 96-well plate. The essential oil concentrations are expressed in term of µg/mL of essential oil. As interaction between volatile compounds between samples could occur, we decided to alternate one test line with two lines filled with culture media. The growth of the parasite was recorded after 48 h of incubation using lactate dehydrogenase (pLDH) activity as parameter according to Makker method [39]. A positive control was used in all the repetitions. This positive control was composed of Artemisinin from Sigma-Aldrich (Machelen, Belgium) at a concentration of 100 µg/mL. Sigmoidal curves allowed the determination of half inhibitory concentration (IC_50_).

### 2.4. Statistical Analysis

#### 2.4.1. Data Analysis

Hierarchical cluster analysis (HCA) and principal component analysis (PCA) (Ward’s method) were used to investigate the seasonal effect on essential oil composition. Analyses on the 19 samples collected during a specific period were performed with Xlstat (Adinsoft, Paris, France).

#### 2.4.2. Biological Activities Analysis

Data were analyzed using IBM SPSS version 20. Results were presented in terms of means. Multiple comparisons of mean values were set up using one-way parametric analysis of variance (ANOVA). The DUNCAN test was used to appreciate the differences between the means at *p*-value ˂ 0.05. The relationship between the different parameters was studied using Pearson correlation.

## 3. Results and Discussion

### 3.1. Chemical Composition of Essential oils and Yields

*Z. leprieurii* organs were collected over a period of seven months for the leaves and trunk barks and five months for the fruits within one single year. Meteorological data were recorded during the collection period (Table 1). The highest rainfall values were recorded in May, June, October and November, with 164.50 mm, 205.70 mm, 310.80 mm and 206.40 mm. In July and August, the rainfall was moderate, with 71.30 mm and 61.70 mm, respectively. The same trend was observed for the temperature. However, temperature variations were low during the collection period, with temperature ranging from 28.40 °C to 25.00 °C. The trends observed for these two variables in addition to those of relative humidity and daylight confirm that the months of May, June, October and November represent rainy season months; while those of July, August and September were dry season months. Results (Table 2, Table 3 and Table 4, in bold) showed that essential oil yields obtained in this study (0.02 to 0.04% (*w/w*) for leaves, 0.86 to 1.20% (*w/w*) for trunk bark and 1.13 to 1.51% (*w/w*) for fruits) were consistent with those found in the literature [18,40]. Essential oil yields seem dependent on meteorological variations, as, for each organ, the highest yields were observed in July and August, the collecting moment when the lowest precipitations and temperatures were recorded. When precipitations increased and temperatures were higher, the lowest essential oil yields were obtained. However, as temperature and yields variations were low in this study, those results should to be confirmed with a longer experiment. Results obtained here, though, are supported by previous studies showing that lower precipitation induces higher essential oil yields [41].

**Figure 1:**
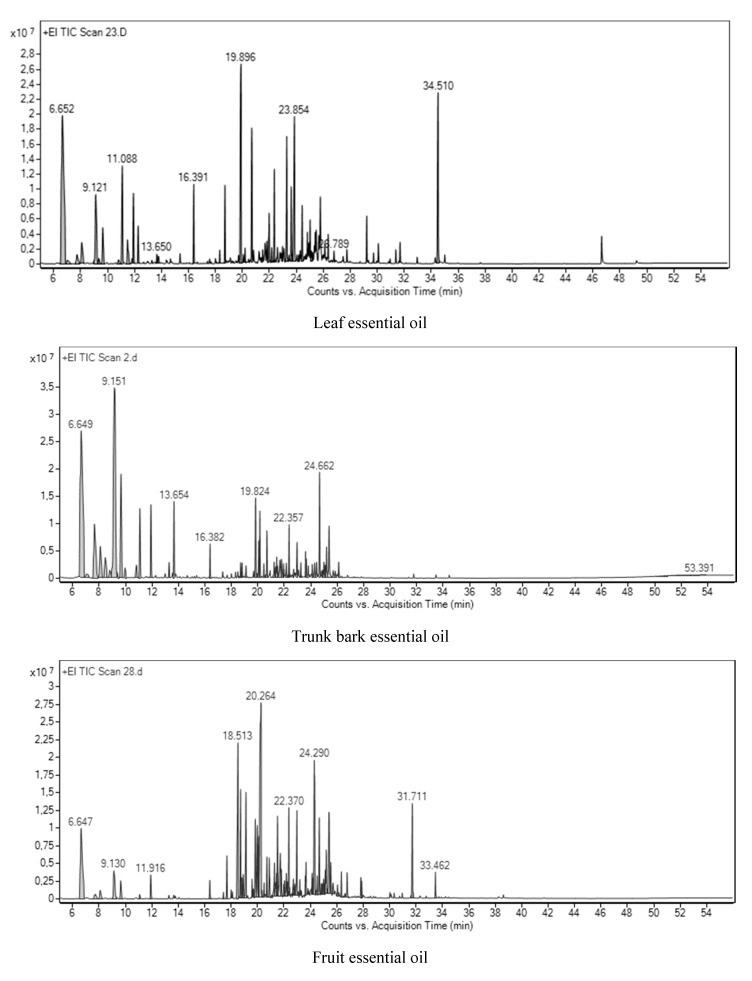
Representative chromatograms for essential oils hydrodistillated from each *Z. leprieurii* organ.

Essential oils hydrodistillated from *Z. leprieurii* organs were analyzed by GC/MS. Representative chromatograms for essential oils hydrodistillated from each organ are presented in Figure 1. Compounds accounting for 97.70–99.50% of global essential oil compositions were identified in the samples. Essential oils hydrodistillated from leaves were dominated by hydrocarbon sesquiterpenes, while methyl ketones were mainly present in trunk bark essential oils. Oxygenated and hydrocarbon monoterpenes were dominant in fruit essential oils. The major compounds identified in these oils were tridecan-2-one and β-caryophyllene in the leaf oils, tridecan-2-one in the trunk bark oils and β-myrcene in the fruit oils.

#### 3.1.1. Leaf Essential Oils

The analysis of essential oils hydrodistillated from leaves allowed for the identification of 42 compounds ranging from 97.70% to 99.50% of the total composition (Table 2). Sesquiterpenes (34.89–70.8%), methylketones (13.10–42.40%) and monoterpenes (4.5–36.18%) were the main components of these essential oils, which were dominated by tridecan-2-one (9.00% to 36.80%) and β-caryophyllene (7.00% to 19.85%). However, the composition of leaf essential oils was not constant over the collecting period, as some compounds that were present in only a minority in some samples were found in higher quantities during certain months. As a first example, there is a drop in tridean-2-one production in June, which is tricky to explain. This is also the case with (*E*)-β-ocimene, whose content was less than 4% from June to November, while in May, it was found to be at 23.57%. Caryophyllene oxide, which represented 5.7–6% of the total oil compositions from June to July, was only found in trace amounts during the other months. Undecan-2-one was also exceptionally present at 8% in May and August. Dendrolasin was present in significant amounts (4–16.4%) in all months except in May. This last molecule has well-known antimicrobial and antibacterial properties, and is also used in the treatment of cancer [42,43,44]. We also noticed the presence of thymol, an oxygenated monoterpene, at 13.30% in August. The chemical compositions of essential oils previously reported from two different Côte d’Ivoire locations [18] collected in February and November 2016 were different to those described in this study. *Z. leprieurii* leave essential oils thus exhibiting various chemotypes: for example, we describe here a chemotype with high proportions of dendrolasin. Moreover, the essential oil composition reported from Nigeria and Cameroon was dominated by limonene (94.90%) [19] and ocimene (91.5%) [40], showing that environmental or genetic factors impact essential oil compositions. Most of the major compounds that were detected in leaf essential oils are already known for their beneficial biological activities, such as insecticidal, antioxidant and anti-inflammatory activities. For example, the β-caryophyllene is a molecule characterized by high antioxidant and anti-inflammatory activities [45].

#### 3.1.2. Trunk Bark Essential Oils

In total, 29 compounds were identified in the seven trunk bark oil samples, accounting for 98.30–99.40% of the whole composition (Table 3). Essential oils hydrodistillated from trunk bark were dominated by tridecan-2-one (45.26–78.80%) and α-humulene, which was also present in a significant content (4.3–12.73%). Hydrocarbon monoterpenes were only present as traces. As for leaf essential oils, the composition of essential oils hydrodistillated from the trunk bark was not consistent during the studied period. Indeed, some sesquiterpenes were only present in high a content during a given period: β-caryophyllene (8.1–13.20%) from May to July and September; tridecan-2-ol was found up to 10.10% in June; and (*E*,*E*)-farnesol (12.5% and 11.1%) in May and July, respectively. This chemical profile shows differences with those reported during our previous work in Côte d’Ivoire. Those differences may be due to the harvesting season and to the harvesting sites, which were not the same in those studies [18]. Moreover, these described compositions are different from those described in Nigeria, in which caryophyllene oxide (23.00%) and humulenol (17.50%) were the major components of trunk bark essential oil [18], showing that the essential oil composition is largely dependent on the plant localization.

In view of the use of tridecan-2-one in the food, pharmaceutical and cosmetic industry [46], trunk bark essential oils of Ivorian *Z. leprieurii* has a high potential. In addition to the major compounds, other minor molecules such as β-caryophyllene and α-humulene were also found in this essential oil, those having interesting antioxidant, anti-inflammatory, antibacterial and insecticidal effects, enhancing the potential use of this essential oil in the pharmaceutical industry [47,48].

#### 3.1.3. Fruit Essential Oils

GC/MS analysis resulted in the identification of 43 constituents of the essential oils hydrodistillated from *Z. leprieurii* fruits (Table 4), accounting for 98.27–99.30% of the total essential oil compositions. This oil was dominated by β-myrcene (16.4–48.27%) but methyl nerate was also present in significant amounts (4.4–6.7%). Moreover, some minor compounds of certain months were present in high quantities in other samples. In particular, citronellol was present at 28.24% in November, but was lower than 6.6% the other months. Furthermore, geranial, which was present in traces in July, saw its content increase in the other months (5.3–6.10%). Some compounds were present in remarkable contents in July: (*E*)-β-ocimene (8.3%); perillene (6.5%); decanal (8.3%); spathulenol (5.2%); and caryophyllene oxide (9.6%).

The essential oils hydrodistillated from *Z. leprieurii* fruits during different months mainly contained monoterpenes hydrocarbons, which is in agreement with the chemical composition of fruit essential oils of the same species studied in Cameroon. Indeed, two different studies conducted in two distinct Cameroon sites showed citronellol (29.90% [20]; 17.37% [17]) and (*E*)-β-ocimene (44% [40]; 90.30% [49]) as the major compounds. Nevertheless, the chemical compositions characterized here are different from those already described. As essential oils obtained here and in previous studies were not hydrodistillated from plants growing in the same place and during the same period, differences in chemical compositions can be explained by climatic and environmental factors depending on each country, while one also cannot exclude a genetic influence that would combine with the other factors of variability.

**Figure 2:**
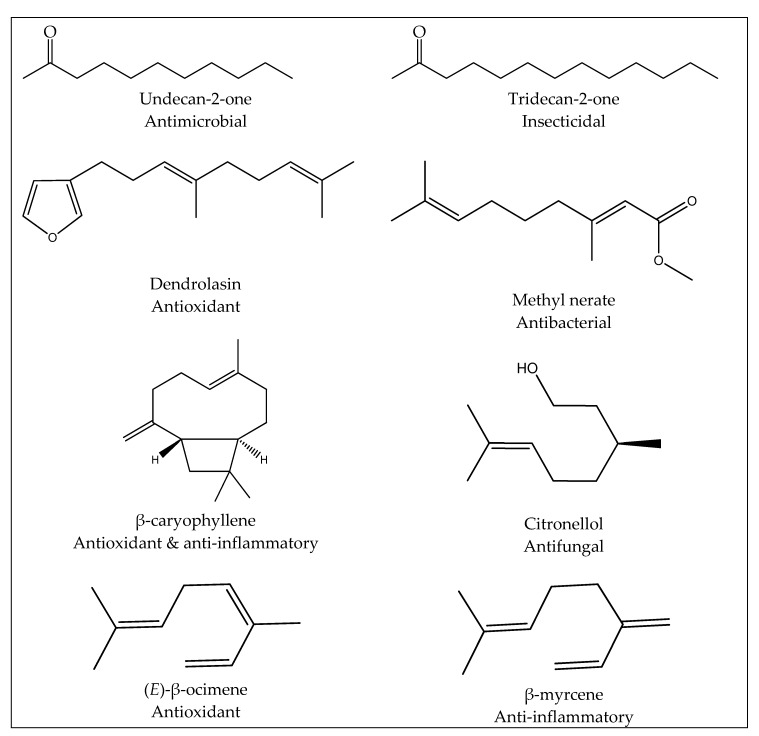
Some major seasonal compounds present in the leaf, trunk bark and fruit essential oils of *Z. leprieurii* from Côte d’Ivoire and their known biological activities [45,50,51,52].

The chemical analysis of essential oils hydrodistillated from the different organs of *Z. leprieurii* from Côte d’Ivoire highlighted the presence of a wide range of compounds, most of them already known for their different interesting biological activities. The presence of those molecules can explain the various uses of *Z. leprieurii* in traditional medicine for the treatment of many different affections, as mentioned above. The main molecules found in Ivorian *Z. leprieurii* essential oils and their known biological properties are presented in Figure 2.

### 3.2. Seasonal Effect on Essential Oil Composition

HCA and PCA analysis were performed to investigate the seasonal effect on essential oil compositions.

**Figure 3:**
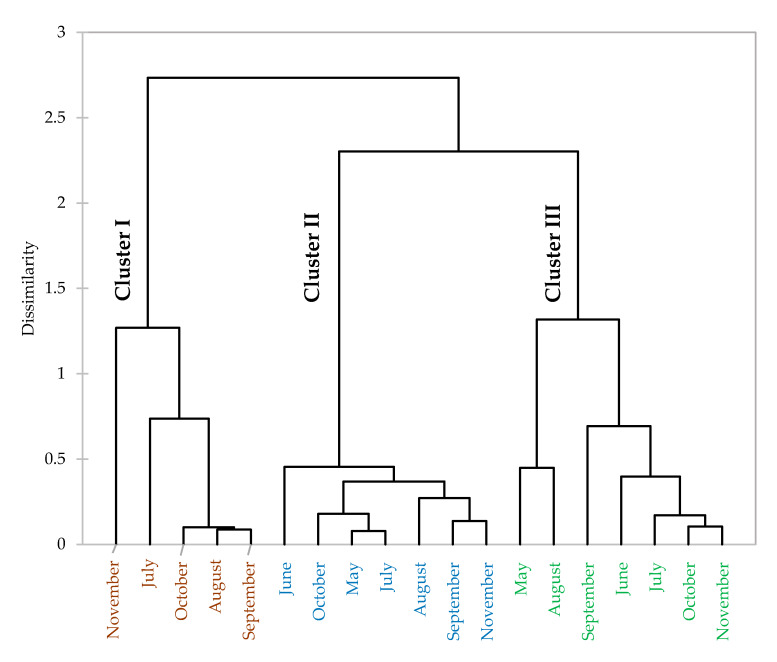
Dendrogram representing *Zanthoxylum leprieurii* essential oil samples. Cluster I: fruits; cluster II: trunk bark; and cluster III: leaves.

The HCA dendrogram (Figure 3), based on the Euclidean distance between collected samples, showed three distinct clusters, each one specific to one plant organ: (i) cluster I for fruits; (ii) cluster II for trunk bark; and (iii) cluster III for leaves. This shows that there is a significant difference in the composition of essential oils hydrodistillated from different *Z. leprieurii* organs. In addition, a seasonal effect was observed among each group, showing variation in the compositions of essential oils hydrodistillated from the same organ during the collection period. This seasonal effect was higher for the leaf essential oils, as the intra-class variance for those samples (2.50) was higher than for the fruit (1.77) and for the trunk bark (0.35) samples. It is possible that this higher variance for leaf essential oil samples is related to the fact that new leaves are produced all year long, while fruits are only produced at certain times of the year and the trunk bark develops very slowly over a period of several months or years. Leaves are then more susceptible to seasonal variations, such as levels of light exposure, state of maturity and water stress, than fruits and the trunk bark. Trunk bark essential oils have a lower chemical variability, probably due to the fact that the trunk bark formation is slow, and thus less impacted by environmental factors [53]. Results are supported by the fact that seasonal differences in the chemical composition of essential oils from fruits, trunk bark and leaves have already been highlighted for other *Zanthoxylum* species [21,23]. Indeed, while the chemical compositions of essential oils are genetically determined, it can be considerably modified by factors such as temperature, light, seasonality, water availability and nutrition. Biosynthesis of different compounds can be induced by environmental stimuli, which can change metabolic pathways [54,55].

**Figure 4:**
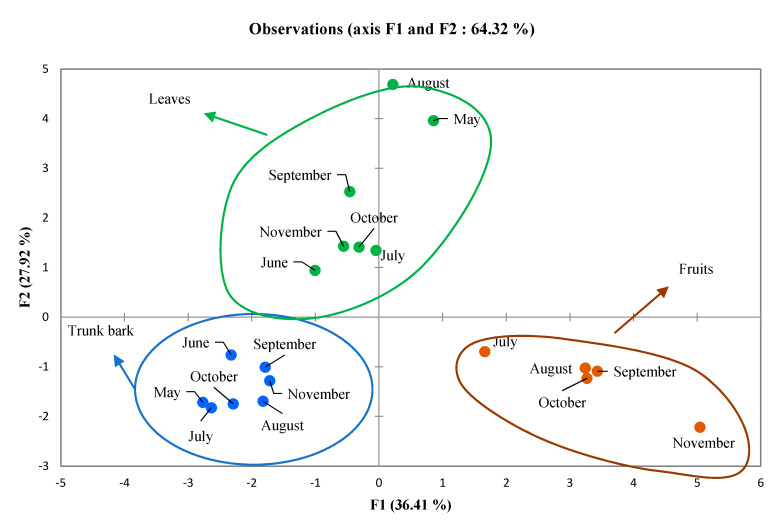
Principal component analysis of the chemical composition of essential oils hydrodistillated from *Zanthoxylum leprieurii* leaves, trunk bark and fruits from Côte d’Ivoire; described according to months and major compounds.

For the PCA analysis, the chemical composition data were projected through linear combinations of the 15 variables that were identified in all samples. Results showed that the first two axes (F1 and F2) explained 64.32% of the total variance (F1: 36.41% and F2: 27.92%). PCA results (Figure 4) showed three different specific clusters, each one being represented by one plant organ. Fruit essential oil samples in cluster I were mainly composed of β-myrcene (36.78 ± 14.67%), citronellol (9.98 ± 10.75%), geraniol (6.14 ± 4.42%), methyl nerate (5.64 ± 0.86%) and geraniol (4.36 ± 2.32%). Cluster II included trunk bark essential oil samples dominated by methylketones with tridecan-2-one (61.56 ± 12.65%) as the principal component. However, α- humulene (7.67 ± 2.71%), β-caryophyllene (6.82 ± 4.25%) and tridecan-2-ol (5.95 ± 2.58%) were also present in significant amounts. Cluster III included the leaf oil samples that were mainly composed of tridecan-2-one (24.11 ± 10.47%), β-caryophyllene (13.97 ± 4.94 %), dendrolasin (8.34 ± 4.72) and α-humulene (4.82 ± 1.29%). This group also showed high levels of (*E*)-β-ocimene (5.35 ± 8.56%), undecan-2-one (4.20 ± 3.34%), linalool (3.73 ± 2.89%), thymol (3.31 ± 4.91%), α-farnesene (3.16 ± 2.98%) and β-elemene (3.13 ± 1.83%).

### 3.3. Essential oil Biological Activities

As mentioned previously, *Z. leprieurii* is widely used in traditional medicine for the treatment of different afflictions. Several authors have already supported those uses by reporting interesting biological properties of essential oils and solvent extracts obtained from this species growing in different places. However, it was shown here that *Z. leprieurii* essential oil chemical composition varies widely depending on the organ of the plant used and depending on the collection month. It is thus important to evaluate the biological activities of the essential oil hydrodistillated in this study, with regards to their compositions. Antioxidant, anti-inflammatory, insecticidal and anti-malarial properties of essential oils hydrodistillated from *Z. leprieurii* growing in Côte d’Ivoire were then evaluated. Essential oils used for the biological activity tests were selected based on their chemical composition. The August-selected leaf essential oil sample was characterized by high amounts of tridecan-2-one (30.20%), β-caryophyllene (13.70%) and thymol (13.30%). The major compounds of the chosen July trunk bark sample were tridecan-2-one (51.40%), (*E*,*E*)-farnesol (11.10%) and β-caryophyllene (8.50%). Finally, the July fruit sample was characterized by high proportions of β-myrcene (16.40%), caryophyllene oxide (9.60%), (*E*)-β-ocimene (8.30%) and decanal (8.30%).

#### 3.3.1. Antioxidant Activity

##### DPPH Free Radical Scavenging Assay

According to Rice-Evans [56], the antioxidant activity of a compound corresponds to its ability to resist oxidation. The free radical scavenging ability of selected essential oils from *Z. leprieurii* leaves, trunk bark and fruits were determined using DPPH with Trolox as a positive control.

The results (Table 5) showed that all essential oil samples were able to reduce the stable DPPH radical to yellow diphenylpicrylhydrazine, with the scavenging effects increasing with higher essential oil concentrations (*p*-value ˂ 0.05). Leaf essential oil had the highest antioxidant activity (IC_50_: 33.12 ± 0.07 µg/mL), followed by trunk bark oil (IC_50_: 65.68 ± 0.12 µg/mL) and fruit oil (IC_50_: 103,55 ± 0.35 µg/mL). The comparison with the Trolox standard (29.13 ± 0.04 µg/mL) showed that the selected leaf essential oil sample has a high antioxidant activity. This activity could be due to the high contents in β-caryophyllene and thymol of this essential oil, as both of those molecules are already known for their antioxidant properties [57]. Those molecules were either present in lower quantities, or absent in the other tested essential oil samples (trunk bark and fruit).

High DPPH free radical scavenging activity was also described in leaf essential oils from other *Zanthoxylum* species; with an IC_50_ value of 27.00 ± 0.1 µg/mL for Indian samples [58]. However, our results strongly differed to those of Tchabong [40], who obtained IC_50_ values of 770 µg/mL and 1800 µg/mL for *Z. leprieurii* fruit and leaf oils from Cameroon, respectively. Those differences in antioxidant properties of essential oil samples from the same species and families collected at different sites and at different periods are probably due to differences in their chemical compositions. Those differences may come from the studied organ, as we showed that essential oil composition variability mainly comes from the chosen organ, but also from genetic factors and/or environmental factors.

##### Ferric-Reducing Antioxidant Power

**Figure 5:**
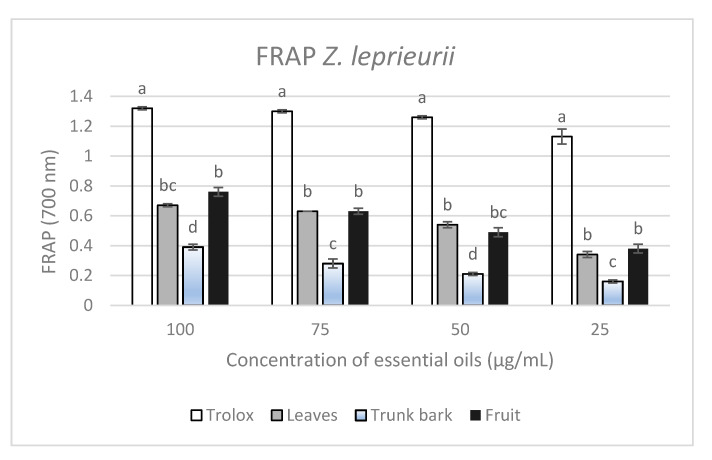
The ferric-reducing power of essential oils hydrodistillated from leaves, trunk bark and fruits of *Z. leprieurii*. Mean values and standard deviation values were presented (*n* = 3). For a same concentration, data with the same letter were not significantly different from each other according to Duncan’s test (*p*-value ˂ 0.05).

The ferric-reducing antioxidant power (FRAP) of essential oils extracted from leaves, trunk bark and fruits of *Z. leprieurii* was studied here for the first time. Results (Figure 5) showed that essential oils exhibited strong antioxidant activities, which were higher with increasing oil concentrations (*p*-value ˂ 0.05) [59]. Fruit and leaf essential oils exhibited higher FRAP activity than trunk bark oils. All these organs were compared to the Trolox, which represented the standard.

Two different assays, DPPH and FRAP, were conducted in this study to evaluate the antioxidant potential of essential oils hydrodistillated from *Z. leprieurii* leaves, trunk bark and fruits. The two different tests resulted in dissimilar results, as leaf and fruit oils gave the highest and the lowest antioxidant activities with the DPPH free radical scavenging assay, respectively; while in the ferric-reducing antioxidant power assay, the highest antioxidant activities were obtained with leaf and fruit oils. Variations in the antioxidant activities of essential oils evaluated by DPPH and FRAP methods are probably due to the differences in reagents used by each method [60]. Indeed, the DPPH assay evaluates the ability of essential oils to scavenge free radicals, while the FRAP method assesses essential oils’ reducing power. The results obtained here showed that essential oils hydrodistillated from different *Z. leprieurii* organs have interesting antioxidant properties, which originate from two different modes of action: free radical scavenging and reducing abilities. The various compounds in essential oils hydrodistillated from *Z. leprieurii* organs are probably the origin of those different antioxidant activities [61,62]. For example, quantities of (*E*)-β-ocimene, perillene and caryophyllene oxide, which are known for their antioxidant properties [63,64], were found in the fruit oil sample, which were present in much lower proportions or completely absent in other essential oils.

#### 3.3.2. Anti-Inflammatory Activity

In order to assess the anti-inflammatory potential of *Z. leprieurii* essential oils, their lipoxygenase inhibitory activity was evaluated, and the anti-denaturation method of bovine albumin serum (BSA) was also used.

##### Lipoxygenase Denaturation Inhibition Activity

The tested essential oils showed high to moderate lipoxygenase inhibitory activity (IC_50_: 26.26 ± 0.04 µg/mL, 28.40 ± 0.02 µg/mL and 32.42 ± 0.15 µg/mL for leaf, trunk bark and fruit oils, respectively) when compared to standard Quercetin (21.57 ± 0.10 µg/mL) (Table 5). These results show that *Z. leprieurii* essential oils have anti-inflammatory properties, as has also been previously described with *Z. leprieurii* growing in different places [65,66].

##### Inhibition of Albumin Denaturation

In vitro anti-inflammatory properties of *Z. leprieurii* trunk bark, leaf and fruit essential oils were evaluated by the anti-denaturation method of bovine albumin serum (BSA) for the first time, in comparison with the control Diclofenac (IC50: 21.90 ± 0.08 µg/mL). The results (Table 5) showed that *Z. leprieurii* leaf, fruit and trunk bark essential oils have high-to-moderate anti-inflammatory activities, with IC_50_ values of 26.08 ± 0.12 µg/mL, 26.68 ± 0.09 µg/mL and 35.07 ± 0.15 µg/mL, respectively. Moreover, the percentage of BSA protection was dependent on essential oil concentrations (*p*-value ˂ 0.05). The origin of these high lipoxygenase inhibitory activities could be the difference in organ content of monoterpenes, methylketones and sesquiterpenes, which are known for their anti-inflammatory activities [67,68,69].

#### 3.3.3. Insecticidal Activity

Losses due to insect infestation during grain storage are a serious problem around the world, and more acutely in developing countries. Consumption of grains is not the only loss caused by insects, as a high level of pest detritus also leads to grains being unfit for human consumption in terms of quality. It could be estimated that one third of the world’s food production is destroyed by insects every year, which represents more than $100 billion. The highest losses occur in developing countries (43%), such as Côte d’Ivoire [70]. In the tropical zone, average losses range from 20% to 30%, while in the temperate zones, losses are from 5% to 10% [71]. Moreover, the trend to use natural insecticides to avoid chemical residues in food is growing.

The insecticidal activities of *Z. leprieurii* trunk bark, leaf and fruit essential oils were evaluated against *Sitophilus granarius*, one of the most damaging pests of stored cereals in the world. This insect is a primary pest, as it is able to drill holes in grains, laying its eggs inside them and allowing secondary pests to develop [33].

Results showed that all essential oils were efficient to kill insects in 24 h, with trunk bark oil showing the highest insecticidal activity (LC_50_ = 8.87 µL/mL) in comparison with leaf and fruit essential oils (LC_50_ = 15.77 µL/mL and 11.26 µL/mL, respectively); those activities were slightly lower than those of the chemical insecticide Talisma UL (LC_50_ = 3.44 µL/mL). Moreover, in comparison with cinnamon (*Cinnamomum zeylanicum*) and clove (*Syzygium aromaticum*) essential oils, generally described as exhibiting high insecticidal activities [72], LC_50_ of *Z. leprieurii* essential oils is lower, showing better insecticidal activities of the latter and thus promising prospects for application in the protection of stored foodstuffs. Concerning LC_90_ and LC_95_, results showed that the chemical insecticide (LC_90_ = 27.83 µL/mL, LC_95_ = 56.66 µL/mL) was less effective than leaf essential oil (LC_90_ = 26.27 µL/mL, LC_95_ = 31.26 µL/mL) and trunk bark essential oil (LC_90_ = 23.69 µL/mL, LC_95_ = 33.10 µL/mL), but more effective than fruit essential oils (LC_90_ = 93.20 µL/mL, LC_95_ = 191.20 µL/mL). These data indicate that the insecticidal effect of essential oils varies depending on the chemical composition and synergistic effects occurring between the compounds [73]. In this study, essential oils hydrodistillated from *Z. leprieurii* organs had an interesting effect on *Sitophilus granarius* adults, as insecticidal activities were better than those of *Z. fagara* and *Z. monoplyllum* (LC_50_ of 153.9 µL/mL and 140.1 µL/mL, respectively) [74]; and the LC_50_ was better than those reported on larvicidal activity [75] with *Z. leprieurii* extracts and *Z. avicennae* essential oil [76]. It should be noted that chemical composition of essential oils is different among these *Zanthoxylum* species and, according to the author [77], mortality evolution showed that toxicity depends on aspects such as the chemical composition and the target insect sensitivity.

The repulsive effect of *Z. leprieurii* trunk bark, leaf and fruit essential oils and chemical insecticides were also evaluated by the McDonald method. The results (Table 6) showed a high repulsive effect for the trunk bark essential oil (88.83%), followed by leaf essential oil (76.66%) and fruit essential oil (61.00%), in comparison with the low repulsive effect of the chemical insecticide Talisma UL (24.78%). Furthermore, repellent properties were dose–response correlated and high when compared to other essential oils considered to be highly repulsive [78].

#### 3.3.4. Anti-Plasmodial Activity

The anti-plasmodial activity of *Z. leprieurii* trunk bark, leaf and fruit essential oils was evaluated here for the first time. The results (Table 5) showed that trunk bark essential oil has a moderate anti-plasmodial activity (IC_50_: 37.49 ± 4.2 µg/mL), and leaf essential oil has a low activity (IC_50_: 59.30 ± 3.4 µg/mL), in comparison with the artemisinin standard (IC_50_: 0.004 ± 0.001 µg/mL). No significant anti-plasmodial activity was highlighted for the fruit essential oil (IC_50_ > 100). The moderate trunk bark anti-plasmodial activity may be due to methylketones, as tridecan-2-one is the dominant compound in this oil. However, no studies have yet shown the anti-plasmodial activity of this molecule. Moreover, it is possible that the highlighted activity comes from the presence of minor compounds, as well as from the synergy between different molecules. Nevertheless, studies were carried out on *Z. leprieurii* and other species of *Z. chalybeum* and *Z. zanthoxyloides* plant extracts, showing high anti-plasmodial activities [79,80,81,82], all of which supports the effective use of *Zanthoxylum* species in traditional medicine for the treatment of malaria.

## 4. Conclusions

In this study, the variability in the chemical composition of leaf, trunk bark and fruit essential oils hydrodistillated from Ivorian *Z. leprieurii* was studied for the first time over seven months for leaves and trunk bark, and five months for fruits. Results showed that essential oils were mainly dominated by sesquiterpenes (β-caryophyllene, dendrolasin and thymol), methylketones (tridecan-2-one and undecane-2 one) and monoterpenes (β-myrcene, (*E*)-β-ocinene and perillene) in leaf, trunk bark and fruit samples, respectively. Statistical PCA and HCA analysis showed that the variability in essential oil compositions mainly comes from the organ, as all samples were clustered in three groups, each one corresponding to one organ. However, differences in essential oil compositions inside each cluster were highlighted, showing the probable impact of the seasonal effect on essential oil compositions. Those differences in essential oil compositions may be due to different seasonal parameters, as it was shown here that the temperature, precipitations and humidity were not constant during the plant collecting period. However, it is also known that biotic factors, such as pest attacks, widely impact essential oil chemical compositions. Those were not recorded during this study, but may also be at the origin of essential oil variability. As a perspective, it would be interesting to study their impact on *Z. leprieurii* essential oil variability. Moreover, the study was conducted with plants growing on the same site. The comparison of the present results with those of the existing literature considering *Z. leprieurii* plants growing in other countries showed totally different essential oil compositions, showing that genetic differences might also induce dissimilar essential oil compositions, resulting in distinct essential oil chemotypes.

*Z. leprieurii* is widely used in traditional medicine for the treatment of different diseases, such as rheumatism, tuberculosis, urinary infections and generalized body pains. In order to explain those uses, in-vitro biological activities of hydrodistillated essential oils were studied here. Results obtained in this study showed strong antioxidant, anti-inflammatory and moderate anti-plasmodial activities. Moreover, expected results also showed high differences in the biological activities of essential oils linked with their differences in chemical composition, which should be taken into account in future research on *Z. leprieurii* essential oil biological activities, but also to find the proper plant harvesting moment for a use in traditional medicine. However, while those results are promising and confirm the relevance of the traditional uses of these plants, in-vitro experiments should be supported by in-vivo tests, as differences can be observed between in-vitro and in-vivo test results.

Grain storage is particularly problematic, as pests cause large losses. Ivoirian essential oils from *Z. leprieurii* demonstrated an interesting repellent effect and contact toxicity properties against *Sitophilus granarius* with the essential oils extracted from the three organs tested (trunk bark, leaves and fruits). All these essential oils are promising candidates for developing new plant insecticides to protect stored products. Moreover, according to De Lucas and colleagues [83], parts of the plant could also be used in silos directly without the extraction step of the essential oils to control pest losses. Indeed, this practice is widely used in Africa because plant material is readily available and usable without any transformation. Moreover, it is easy to separate the plant material added to the silo from the grain for use as food or feed. It would then be interesting to study the insecticidal properties of *Z. leprieurii* organs in that way.

In conclusion, *Z. leprieurii* from Côte d’Ivoire as a medicinal and aromatic plant provides interesting sources of biologically active compounds, such as antioxidants, anti-inflammatory agents and natural insecticides. The results obtained here support the current uses of this plant in traditional medicine, but also highlight the importance of the location and the season on chemical composition and thus biological properties.

## Figures and Tables

**Table 1:** Meteorological parameters recorded during the collection period of *Zanthoxylum leprieurii* organs.

Months	Rainfall (mm)	Relative Humidity (%)	Daylight (h)	Temperature (°C)
May	164.50	80.00	196.30	28.40
June	205.70	85.00	101.60	26.80
July	71.30	84.00	121.00	25.80
August	61.70	84.00	134.00	25.00
September	102.40	81.00	124.20	25.90
October	310.80	84.00	180.40	27.10
November	206.40	78.00	214.40	27.50

Source: SODEXAM (Société d’Exploitation et de Développement Aéronautique, Aéroportuaire et Météorologique), 2017.

**Table 2:** Chemical composition of essential oils hydrodistillated from *Zanthoxylum leprieurii* leaves. Each analysis was realized in triplicate.

						Leaves						
N°	Compounds	Cas Number	Identification	Ria	Rib	May	June	July	Aug	Sep	Oct	Nov
1	α-pinene	80-56-8	MS, RI, STD	931	929	2.48 ± 0.13	8.45 ± 0.57	0.30 ± 0.11	0.60 ± 0.35	0.70 ± 0.15	0.50 ± 0.27	0.30 ± 0.17
2	ß-myrcene	123-35-3	MS, RI, STD	987	988	0.71 ± 0.03	-	5.20 ± 0.21	0.90 ± 0.15	3.40 ± 0.37	0.4 ± 0.31	0.70 ± 0.09
3	p-cymene	25155-15-1	MS, RI, STD	1022	1022	1.48 ± 0.03	-	0.10 ± 0.01	0.30 ± 0.03	-	0.40 ± 0.57	-
4	limonene	138-86-3	MS, RI, STD	1023	1027	-	0.90 ± 0.29	0.40 ± 0.28	-	-	-	-
5	*(E*)-ß-ocimene	13877-91-3	MS, RI, STD	1041	1046	23.57 ± 047	2.60 ± 0.99	3.90 ± 0.84	2.50 ± 0.04	1.30 ± 0.50	2.30 ± 1.91	-
6	γ-terpinene	99-85-4	MS, RI, STD	1060	1057	0.28 ± 0.03	-	-	-	-	-	-
7	linalool	78-70-6	MS, RI, STD	1094	1098	6.44 ± 0.13	0.50 ± 0.34	1.50 ± 0.55	8.50 ± 0.09	1.70 ± 0.23	4.30 ± 0.95	3.20 ± 0.09
8	alloocimene	673-84-7	MS, RI	1128	1129	1.22 ± 0.04	0.40 ± 0.13	0.20 ± 0.10	-	-	-	-
9	terpineol	98-55-5	MS, RI, STD	1190	1191	-	-	0.20 ± 0.03	-	-	-	-
10	decanal	112-31-2	MS, RI, STD	1191	1204	-	-	1.50 ± 0.52	-	-	-	-
11	citronellol	106-22-9	MS, RI, STD	1225	1227	-	-	2.00 ± 0.48	-	1.50 ± 0.27	-	0.30 ± 0.02
12	geraniol	106-24-1	MS, RI, STD	1250	1253	-	-	1.40 ± 0.53	-	1.00 ± 0.74	-	-
13	decyl alcohol	112-30-1	MS, RI, STD	1262	1263	-	-	1.80 ± 0.92	-	-	-	-
14	thymol	89-83-8	MS, RI, STD	1286	1291	-	2.20 ± 0.96	3.10 ± 0.43	13.30 ± 0.31	4.10 ± 0.22	-	-
15	undecan-2-one	112-12-9	MS, RI, STD	1288	1293	8.51 ± 0.35	1.90 ± 0.50	1.20 ± 0.03	8.40 ± 0.12	2.70 ± 0.56	3.20 ± 1.06	2.10 ± 0.98
16	undecan-2-ol	1653-30-1	MS, RI, STD	1298	1300	-	-	0.20 ± 0.04	0.40 ± 0.04	-	-	-
17	methyl nerate	1862-61-9	MS, RI, STD	1319	1323	-	-	2.20 ± 0.74	-	0.80 ± 0.44	-	-
18	δ-elemene	20307-84-0	MS, RI	1334	1339	-	1.20 ± 0.69	0.20 ± 0.00	-	-	-	-
19	α-copaene	3856-25-5	MS, RI, STD	1376	1378	-	0.60 ± 0.23	0.30 ± 0.08	-	0.60 ± 0.11	0.20 ± 0.07	0.40 ± 0.04
20	ß-elemene	515-13-9	MS, RI	1388	1394	3.92 ± 0.06	-	2.70 ± 0.83	4.20 ± 0.07	5.90 ± 0.26	2.30 ± 0.10	2.90 ± 0.20
21	β-caryophyllene	87-44-5	MS, RI, STD	1419	1423	13.51 ± 0.11	18.90 ± 0.48	15.60 ± 0.54	13.70 ± 0.1	19.85 ± 0.82	7.00 ± 1.02	8.60 ± 0.62
22	cadina-4(14),5-diene	54324-03-7	MS, RI	1430	1433	1.83 ± 0.02	0.90 ± 0.26	0.70 ± 0.16	0.30 ± 0.02	2.40 ± 0.81	1.70 ± 0.26	1.50 ± 0.19
23	γ-elemene	3242-08-8	MS, RI	1432	1435	1.19 ± 0.01	4.40 ± 0.45	1.10 ± 0.21	1.10 ± 0.04	3.10 ± 0.06	0.8 ± 0.12	1.10 ± 0.01
24	α-humulene	6753-98-6	MS, RI, STD	1456	1457	3.92 ± 0.02	6.70 ± 0.62	4.50 ± 0.98	3.60 ± 0.04	6.10 ± 0.27	4.10 ± 0.58	4.20 ± 0.32
25	alloaromadendrene	25246-27-9	MS, RI	1457	1465	-	0.50 ± 0.21	0.30 ± 0.06	-	0.50 ± 0.11	0.40 ± 0.06	0.20 ± 0.01
26	germacrene D	23986-74-5	MS, RI, STD	1482	1485	1.96 ± 0.17	0.90 ± 0.50	0.40 ± 0.10	-	1.60 ± 0.40	1.40 ± 0.21	1.00 ± 0.14
27	ß-ionone	14901-07-6	MS, RI, STD	1482	1488	-	-	0.40 ± 0.12	-	-	-	0.20 ± 0.05
28	ß-selinene	17066-67-0	MS, RI	1483	1490	0.32 ± 0.01	1.10 ± 0.13	0.70 ± 0.14	0.80 ± 0.01	1.60 ± 0.30	0.20 ± 0.21	0.50 ± 0.05
29	tridecan-2-one	593-08-8	MS, RI, STD	1487	1495	18.74 ± 0.57	9.00 ± 0.02	22.50 ± 0.98	30.20 ± 0.39	15.80 ± 0.84	36.80 ± 0.06	33.70 ± 0.36
30	selina-4(14),7(11)-diene	515-17-3	MS, RI	1495	1498	-	1.30 ± 0.31	0.80 ± 0.15	1.50 ± 0.90	2.90 ± 0.86	-	-
31	tridecan-2-ol	1653-31-2	MS, RI, STD	1495	1501	-	2.00 ± 0.82	1.70 ± 1.17	-	2.30 ± 0.29	2.40 ± 0.80	3.20 ± 0.33
32	(*3E,6E*)-α-farnesene	502-61-4	MS, RI	1499	1509	3.22 ± 0.29	0.60 ± 0.21	1.20 ± 0.34	0.90 ± 0.07	2.50 ± 0.97	9.10 ± 0.82	4.60 ± 0.55
33	γ-cadinene	39029-4-9	MS, RI	1513	1517	0.42 ± 0.06	1.10 ± 0.36	0.60 ± 0.08	0.20 ± 0.04	1.00 ± 0.21	1.20 ± 0.26	1.30 ± 0.02
34	δ-cadinene	483-76-1	MS, RI	1524	1526	2.25 ± 0.09	4.20 ± 1.01	2.20 ± 0.09	1.00 ± 0.02	4.30 ± 0.13	3.40 ± 0.49	4.30 ± 0.33
35	elemol	639-99-6	MS, RI, STD	1547	1552	0.28 ± 0.02	0.60 ± 0.22	0.20 ± 0.01	0.30 ± 0.01	0.30 ± 0.05	0.40 ± 0.03	1.20 ± 0.09
36	nerolidol	7212-44-4	MS, RI, STD	1557	1564	-	1.50 ± 0.30	1.10 ± 0.39	1.00 ± 0.03	1.60 ± 0.49	4.80 ± 0.22	3.40 ± 0.32
37	dendrolasin	23262-34-2	MS, RI	1576	1580	1.79 ± 0.08	8.60 ± 0.95	9.40 ± 0.90	4.00 ± 0.06	7.60 ± 0.09	10.60 ± 0.44	16.40 ± 0.85
38	spathulenol	6750-60-3	MS, RI	1578	1582	-	1.50 ± 0.46	1.10 ± 0.03	-	-	-	0.40 ± 0.00
39	caryophyllene oxide	1139-30-6	MS, RI, STD	1583	1588	-	6.00 ± 0.83	5.70 ± 0.79	-	-	-	-
40	ζ-cadinol	5937-11-1	MS, RI	1639	1645	-	1.10 ± 0.08	0.60 ± 0.03	-	0.70 ± 0.21	0.60 ± 0.15	0.80 ± 0.04
41	α-cadinol	481-34-5	MS, RI	1650	1659	0.28 ± 0.03	1.30 ± 0.11	0.70 ± 0.01	-	1.10 ± 0.44	0.80 ± 0.23	0.90 ± 0.09
42	pentadecanal	2765-11-9	MS, RI	1713	1715	-	2.10 ± 0.28	-	-	-	-	1.60 ± 0.79
	Monoterpene hydrocarbons (%)					29.74	12.85	10.10	4.30	5.40	3.60	1.00
	Oxygenated monoterpene (%)					6.44	2.70	11.50	21.80	8.20	4.30	3.50
	Sesquiterpene hydrocarbons (%)					32.54	48.10	30.90	27.30	52.35	31.80	30.60
	Oxygenated sesquiterpenes (%)					2.35	22.70	18.10	5.30	12.10	17.20	25.40
	Others (%)					27.25	13.10	28.90	39.00	20.80	42.40	38.50
	Identified compounds (%)					98.32	99.45	99.50	97.70	98.85	99.30	99.00
	**Yield (%)**					**0.02**	**0.02**	**0.03**	**0.04**	**0.02**	**0.02**	**0.02**

Identification methods: RIa, theoretical kovats indices (Pubchem and NIST); RIb, calculated kovats indices; MS, mass spectra comparison with PAL 600^®^libraries; STD, retention time and mass spectra comparison with commercially available standards; RI, retention index comparison with the literature; CAS number; -, Under perception threshold.

**Table 3:** Chemical composition of essential oils hydrodistillated from *Zanthoxylum leprieurii* trunk bark. Each analysis was realized in triplicate.

						Trunk Bark						
N°	Compounds	Cas Number	Identification	Ria	Rib	May	June	July	Aug	Sep	Oct	Nov
1	α-pinene	80-56-8	MS, RI, STD	931	929	0.52 ± 0.03	-	-	-	-	-	-
2	ß-myrcene	123-35-3	MS, RI, STD	987	988	-	-	-	-	1.00 ± 0.14	-	-
3	citronellal	106-23-0	MS, RI, STD	1153	1153	-	-	-	-	1.50 ± 0.08	-	-
4	citronellol	106-22-9	MS, RI, STD	1225	1227	-	-	-	-	1.60 ± 0.08	-	-
5	geraniol	106-24-1	MS, RI, STD	1250	1253	-	-	-	-	1.50 ± 0.14	-	-
6	thymol	89-83-8	MS, RI, STD	1286	1291	-	5.10 ± 0.36	0.50 ± 0.08	-	1.30 ± 0.05	-	4.10 ± 0.50
7	ß-elemene	515-13-9	MS, RI	1388	1394	-	0.60 ± 0.15	-	-	1.30 ± 0.03	-	-
8	α-bergamotene	17699-05-7	MS, RI	1415	1417	4.43 ± 0.10	0.50 ± 0.27	3.20 ± 0.69	1.60 ± 1.12	0.70 ± 0.01	1.50 ± 0.56	4.80 ± 0.60
9	β-caryophyllene	87-44-5	MS, RI, STD	1419	1423	9.51 ± 0.37	13.20 ± 0.33	8.50 ± 0.35	2.10 ± 0.13	8.10 ± 0.08	4.10 ± 0.79	1.80 ± 0.23
10	γ-elemene	3242-08-8	MS, RI	1432	1435	-	0.60 ± 0.21	0.10 ± 0.01	-	-	-	-
11	geranylacetone	3796-70-1	MS, RI	1455	1453	-	1.10 ± 0.51	0.50 ± 0.19	-	0.40 ± 0.02	0.30 ± 0.06	-
12	α-humulene	6753-98-6	MS, RI, STD	1456	1457	12.73 ± 1.41	4.30 ± 1.09	7.70 ± 0.62	6.20 ± 0.94	7.40 ± 0.11	8.10 ± 1.01	6.30 ± 0.03
13	α-curcumene	644-30-4	MS, RI	1482	1484	0.83 ± 0.18	-	0.70 ± 0.10	0.30 ± 0.11	-	0.40 ± 0.13	1.30 ± 0.08
14	ß-selinene	17066-67-0	MS, RI	1483	1490	-	0.30 ± 0.14	0.10 ± 0.01	-	0.30 ± 0.01	-	0.25 ± 0.33
15	tridecan-2-one	593-08-8	MS, RI, STD	1487	1495	45.26 ± 0.96	56.30 ± 0.31	51.4 ± 1.15	78.80 ± 0.55	54.40 ± 0.56	70.2 ± 0.95	71.36 ± 0.70
16	tridecan-2-ol	1653-31-2	MS, RI, STD	1495	1501	2.23 ± 0.17	10.10 ± 0.61	6.25 ± 0.17	4.30 ± 1.43	5.70 ± 0.20	6.40 ± 0.04	4.20 ± 0.40
17	(*3E,6E*)-α-farnesene	502-61-4	MS, RI	1503	1509	3.07 ± 0.28	1.20 ± 0.03	1.70 ± 0.04	2.70 ± 0.12	2.50 ± 0.09	2.30 ± 0.21	1.70 ± 0.03
18	ß-bisabolene	495-61-4	MS, RI	1505	1511	1.30 ± 0.05	-	1.20 ± 0.24	0.60 ± 0.37	-	0.50 ± 0.06	1.37 ± 0.20
19	γ-cadinene	39029-4-9	MS, RI	1513	1517	-	-	0.20 ± 0.02	-	0.60 ± 0.08	-	0.2 ± 0.08
20	δ-cadinene	483-76-1	MS, RI	1524	1526	-	0.20 ± 0.04	0.30 ± 0.04	0.10 ± 0.03	1.20 ± 0.05	-	0.38 ± 0.23
21	elemol	639-99-6	MS, RI, STD	1547	1552	-	0.30 ± 0.15	-	-	2.90 ± 0.07	-	0.31 ± 0.30
22	nerolidol	7212-44-4	MS, RI, STD	1557	1564	3.13 ± 0.27	0.40 ± 0.16	1.80 ± 0.63	0.40 ± 0.11	0.70 ± 0.03	1.20 ± 0.35	0.60 ± 0.03
23	caryophyllene oxide	1139-30-6	MS, RI, STD	1583	1588	-	0.20 ± 0.11	0.30 ± 0.00	-	-	-	-
24	ζ-cadinol	5937-11-1	MS, RI	1639	1645	-	-	-	-	0.30 ± 0.02	-	-
25	α-cadinol	481-34-5	MS, RI	1650	1659	-	-	-	-	0.50 ± 0.03	-	-
26	pentadecan-2-one	2345-28-0	MS, RI, STD	1696	1697	0.48 ± 0.02	0.50 ± 0.23	0.70 ± 0.02	-	-	-	-
27	(*E*,*E*)-farnesol	106-28-5	MS, RI	1722	1723	12.5 ± 0.85	3.00 ± 0.96	11.1 ± 0.15	1.40 ± 0.90	2.20 ± 0.45	1.90 ± 0.28	-
28	farnesal	19317-11-4	MS, RI	1738	1744	1.36 ± 0.02	0.40 ± 0.15	1.20 ± 0.05	-	0.30 ± 0.07	0.20 ± 0.04	-
29	methyl farnesoate	3675-00-1	MS, RI	1779	1785	1.90 ± 0.10	1.00 ± 0.35	1.60 ± 0.17	-	0.90 ± 0.08	0.70 ± 0.14	-
	Monoterpene hydrocarbons (%)					0.52	0.00	0.00	0.00	1.00	0.00	0.00
	Oxygenated monoterpene (%)					0.00	5.30	0.50	0.00	5.90	0.00	4.10
	Sesquiterpene hydrocarbons (%)					31.87	21.80	24.20	13.60	22.20	16.90	18.20
	Oxygenated sesquiterpenes (%)					19.37	5.80	16.70	1.80	8.20	4.30	1.10
	Others (%)					47.46	66.40	57.65	83.10	62.10	77.10	75.60
	Identified compounds (%)					99.22	99.30	99.05	98.50	99.40	98.30	99.00
	**Yield (%)**					**0.88**	**0.91**	**1.18**	**1.20**	**0.86**	**0.89**	**0.86**

Identification methods: RIa, theoretical kovats indices (Pubchem and NIST); RIb, calculated kovats indices; MS, mass spectra comparison with PAL 600^®^libraries; STD, retention time and mass spectra comparison with commercially available standards; RI, retention index comparison with the literature; CAS number; -, Under perception threshold.

**Table 4:** Chemical composition of essential oils hydrodistillated from *Zanthoxylum leprieurii* fruits. Each analysis was realized in triplicate.

								Fruits		
N°	Compounds	Cas Number	Identification	Ria	Rib	July	Aug	Sep	Oct	Nov
1	α-pinene	80-56-8	MS, RI, STD	931	929	2.30 ± 0.90	0.90 ± 0.12	0.80 ± 0.04	1.90 ± 0.66	0.60 ± 0.13
2	ß-myrcene	123-35-3	MS, RI, STD	987	988	16.40 ± 0.91	44.80 ± 0.13	46.30 ± 0.10	48.27 ± 0.26	24.7 ± 0.97
3	α-terpinene	99-86-5	MS, RI, STD	1008	1017	-	-	-	-	0.30 ± 0.06
4	p-cymene	25155-15-1	MS, RI, STD	1022	1022	0.20 ± 0.08	0.70 ± 0.09	0.40 ± 0.04	0.30 ± 0.05	0.80 ± 0.04
5	limonene	138-86-3	MS, RI, STD	1023	1027	1.10 ± 0.21	2.60 ± 0.29	2.00 ± 0.07	2.20 ± 0.16	6.00 ± 0.42
6	(*E*)-ß-ocimene	13877-91-3	MS, RI, STD	1041	1046	8.30 ± 0.29	2.00 ± 0.12	1.50 ± 0.08	1.50 ± 0.06	2.0 ± 0.15
7	γ-terpinene	99-85-4	MS, RI, STD	1060	1057	-	-	-	-	0.20 ± 0.01
8	linalool	78-70-6	MS, RI, STD	1094	1098	-	2.50 ± 0.27	3.40 ± 0.12	2.10 ± 0.10	4.20 ± 0.6
9	perillene	539-52-6	MS, RI, STD	1094	1100	6.50 ± 0.12	-	-	-	-
10	alloocimene	673-84-7	MS, RI	1128	1129	0.50 ± 0.07	0.50 ± 0.05	0.50 ± 0.04	0.40 ± 0.05	0.30 ± 0.02
11	isopulegol	7786-67-6	MS, RI	1140	1145	-	-	-	-	0.60 ± 0.15
12	citronellal	106-23-0	MS, RI, STD	1153	1153	0.20 ± 0.01	1.00 ± 0.11	1.40 ± 0.13	0.90 ± 0.12	5.70 ± 0.43
13	limonene oxide	1195-92-2	MS, RI	1175	1182	-	-	0.90 ± 0.01	-	0.90 ± 0.24
14	terpineol	98-55-5	MS, RI, STD	1190	1191	-	-	-	-	0.20 ± 0.05
15	decanal	112-31-2	MS, RI, STD	1202	1204	8.30 ± 0.17	1.80 ± 0.18	2.20 ± 0.02	1.60 ± 0.14	0.70 ± 0.24
16	citronellol	106-22-9	MS, RI, STD	1225	1227	1.90 ± 0.02	6.20 ± 0.55	6.60 ± 0.14	6.30 ± 0.63	28.24 ± 0.10
17	geraniol	106-24-1	MS, RI, STD	1250	1253	1.20 ± 0.03	5.50 ± 0.59	6.20 ± 0.22	6.30 ± 0.51	2.33 ± 0.74
18	decyl alcohol	112-30-1	MS, RI, STD	1262	1263	4.50 ± 0.18	-	-	-	-
19	geranial	141-27-5	MS, RI, STD	1268	1270	-	5.30 ± 0.53	7.60 ± 0.12	5.30 ± 0.31	12.50 ± 0.47
20	undecan-2-one	112-12-9	MS, RI, STD	1288	1293	0.20 ±0.03	-	-	-	-
21	undecanal	112-44-7	MS, RI, STD	1305	1306	0.20 ± 0.02	-	-	-	-
22	methyl nerate	1862-61-9	MS, RI, STD	1319	1323	6.70 ± 0.23	5.7 ± 0.6	4.40 ± 0.15	5.30 ± 0.13	6.10 ± 0.50
23	δ-elemene	20307-84-0	MS, RI	1334	1339	-	-	-	0.10 ± 0.02	-
24	α-cubebene	17699-14-8	MS, RI, STD	1349	1351	-	0.20 ± 0.01	0.10 ± 0.00	0.10 ± 0.01	-
25	α-copaene	3856-25-5	MS, RI, STD	1376	1378	0.60 ± 0.01	0.20 ± 0.02	0.20 ± 0.01	0.20 ± 0.02	-
26	ß-elemene	515-13-9	MS, RI	1388	1394	1.20 ± 0.04	2.10 ± 0.21	1.50 ± 0.01	1.80 ± 0.07	0.20 ± 0.1
27	β-caryophyllene	87-44-5	MS, RI, STD	1419	1423	5.80 ± 0.18	4.90 ± 0.55	4.10 ± 0.03	3.50 ± 0.06	0.50 ± 0.23
28	cadina-4(14),5-diene	54324-03-7	MS, RI	1430	1433	0.80 ± 0.06	1.80 ± 0.20	1.60 ± 0.03	1.30 ± 0.07	-
29	γ-elemene	3242-08-8	MS, RI	1432	1435	0.40 ± 0.02	1.10 ± 0.11	0.90 ± 0.01	0.90 ± 0.05	-
30	α-humulene	6753-98-6	MS, RI, STD	1456	1457	3.70 ± 0.21	1.90 ± 0.21	1.60 ± 0.01	1.50 ± 0.07	0.20 ± 0.09
31	alloaromadendrene	25246-27-9	MS, RI	1457	1465	0.90 ± 0.04	0.30 ± 0.03	0.10 ± 0.01	0.20 ± 0.08	-
32	germacrene D	23986-74-5	MS, RI, STD	1482	1485	0.50 ± 0.04	0.80 ± 0.10	1.10 ± 0.12	1.40 ± 0.18	0.20 ± 0.1
33	ß-selinene	17066-67-0	MS, RI	1483	1490	-	-	0.10 ± 0.02	0.10 ± 0.04	-
34	α-selinene	473-13-2	MS, RI	1488	1498	-	-	0.40 ± 0.01	0.40 ± 0.09	-
35	γ-cadinene	39029-4-9	MS, RI	1513	1517	0.90 ± 0.06	1.10 ± 0.08	0.70 ± 0.01	0.80 ± 0.05	0.20 ± 0.07
36	δ-cadinene	483-76-1	MS, RI	1524	1526	2.70 ± 0.14	3.30 ± 0.35	2.20 ± 0.02	2.70 ± 0.05	0.60 ± 0.15
37	α-calacorene	21391-99-1	MS, RI	1542	1547	0.50 ± 0.01	-	-	-	-
38	elemol	639-99-6	MS, RI, STD	1547	1552	0.10 ± 0.02	0.40 ± 0.04	0.20 ± 0.02	-	-
39	spathulenol	6750-60-3	MS, RI	1578	1582	5.20 ± 0.06	-	-	-	-
40	caryophyllene oxide	1139-30-6	MS, RI, STD	1583	1588	9.60 ± 0.29	-	-	-	-
41	ζ-cadinol	5937-11-1	MS, RI	1639	1645	1.20 ± 0.31	0.30 ± 0.06	0.10 ± 0.01	0.40 ± 0.04	-
42	α-cadinol	481-34-5	MS, RI	1650	1659	1.80 ± 0.24	0.50 ± 0.05	-	0.70 ± 0.08	-
43	cadalene	483-78-3	MS, RI	1678	1679	-	-	-	0.10 ± 0.02	-
Monoterpene hydrocarbons (%)						28.80	51.50	51.50	54.17	34.90
Oxygenated monoterpene (%)						9.80	20.50	26.10	21.30	54.67
Sesquiterpene hydrocarbons (%)						18.10	17.70	14.60	15.10	1.90
Oxygenated sesquiterpenes (%)						25.10	6.90	4.70	6.90	6.10
Others (%)						17.50	1.80	2.20	1.60	0.70
Identified compounds (%)						99.30	98.40	99.10	99.07	98.27
**Yield (%)**						**1.42**	**1.51**	**1.22**	**1.13**	**1.14**

Identification methods: RIa, theoretical kovats indices (Pubchem and NIST); RIb, calculated kovats indices; MS, mass spectra comparison with PAL 600^®^libraries; STD, retention time and mass spectra comparison with commercially available standards; RI, retention index comparison with the literature; CAS number; -, Under perception threshold.

**Table 5:** Biological properties of essential oils hydrodistillated from different *Z. leprieurii* organs. DPPH: 2,2-diphenyl-1-picrylhydrazyl, LOX: lipoxygenase, BSA: bovine serum albumin.

Organs and Standards	Biological Activities IC_50_ (µL/mL)			
	DPPH	LOX Denaturation	BSA Denaturation	Anti-Plasmodial
Leaves	33.12 ± 0.07	26.26 ± 0.04	26.08 ± 0.12	62.3 ± 3.4
Trunk Bark	65.68 ± 0.12	28.40 ± 0.02	35.07 ± 0.15	36.29 ± 4.2
Fruits	103.55 ± 0.35	32.42 ± 0.15	26.68 ± 0.09	>100
Trolox	28.13 ± 0.04			
Quercetin		21.57 ± 0.10		
Diclofenac			21.90 ± 0.08	
Artemisinin				0.004 ± 0.001

**Table 6:** Repulsion percentage of *Sitophilus granarius* after 2 h of treatment with essential oils and Talisma UL. Effect of substance tested [35].

Tested Substances	Average Repulsion (%)	Class	Effect of Substance Tested
Leaf essential oil	76.66	IV	Repulsive
Trunk bark essential oil	88.83	V	Highly repulsive
Fruit essential oil	61.00	III	Mildly repulsive
Talisma UL	24.78	II	Weakly repulsive
